# Mapping marine debris encountered by albatrosses tracked over oceanic waters

**DOI:** 10.1038/s41598-021-90417-x

**Published:** 2021-05-25

**Authors:** Bungo Nishizawa, Jean-Baptiste Thiebot, Fumio Sato, Naoki Tomita, Ken Yoda, Rei Yamashita, Hideshige Takada, Yutaka Watanuki

**Affiliations:** 1 National Institute of Polar Research, 10‑3 Midori‑cho, Tachikawa, Tokyo 190‑8518 Japan; 2 Yamashina Institute for Ornithology, Konoyama 115, Abiko, Chiba 270‑1145 Japan; 3 Graduate School of Environmental Studies, Nagoya University, Furo-cho, Chikusa-ku, Nagoya, 464-8601 Japan; 4 Atmosphere and Ocean Research Institute, The University of Tokyo, 5-1-5 Kashiwanoha, Kashiwa, Chiba 277-8564 Japan; 5 Laboratory of Organic Geochemistry, Tokyo University of Agriculture and Technology, Fuchu, Tokyo 183‑8509 Japan; 6 Graduate School of Fisheries Sciences, Hokkaido University, 3‑1‑1, Minato-cho, Hakodate, Hokkaido 041‑8611 Japan

**Keywords:** Behavioural ecology, Conservation biology, Marine biology, Environmental impact, Animal behaviour

## Abstract

Anthropogenic marine debris is a threat to marine organisms. Understanding how this debris spatially distributes at sea and may become associated with marine wildlife are key steps to tackle this current issue. Using bird-borne GPS- and video-loggers on 13 black-footed albatrosses *Phoebastria nigripes* breeding in Torishima, Japan, we examined the distribution of large floating debris in the Kuroshio Current area, western North Pacific. A total of 16 floating debris, including styrofoam (n = 4), plastic pieces (n = 3), plastic sheet (n = 1), fishery-related items (rope or netting, n = 4), and unidentified debris (n = 4), were recorded across the 9003 km covered by nine birds. The debris was concentrated in the southern area of the Kuroshio Current, where the surface current was weak, and the albatrosses were foraging. The albatrosses displayed changes in flight direction towards the debris when at a mean distance of 4.9 km, similarly to when approaching prey, and one bird was observed pecking at a plastic sheet; indicating that albatrosses actively interacted with the debris. This paper shows the usefulness of studying wide-ranging marine predators through the use of combined biologging tools, and highlights areas with increased risk of debris exposure and behavioral responses to debris items.

## Introduction

The abundance of anthropogenic marine debris is increasing globally, with debris now found in the most remote areas of the open ocean^1,2^, polar seas^3,4^, and the world’s deepest seafloor^5,6^. On a global scale, non-degradable plastic accounts for 73% of marine debris^7^. It was estimated that over 8 million tons of plastic waste from land enter the oceans annually, and the cumulative quantity of plastic in the ocean is predicted to accelerate rapidly^8^.

Marine debris has adverse impacts on marine organisms in several ways^9^. First, entanglement in fishing gear such as lines and netting likely results in sudden death for marine wildlife, by drowning or suffocation^10,11^. Plastic ingestion also causes injury of the digestive tract and inhibits food digestion^12–14^. Further, plastic-derived toxic chemicals from ingested plastic are transported into the organism’s tissues^15–17^, as evidenced in a variety of marine species^18,19^. Besides, marine debris floating at the sea surface provides new habitat surfaces for diverse organisms including seaweeds, barnacles and crustaceans, and spawning substrate for fish and cephalopods^20,21^. Thus, marine debris can also facilitate the dispersal of associated organisms, and hence potentially contribute to biological invasions of non-indigenous species^20,21^.

Monitoring the abundance and distribution of debris at sea has thus far relied on techniques with heavy logistics including direct observation (for the larger-sized debris) and net tows (for smaller items) conducted from ships or aircrafts^22–26^. However, these at-sea surveys are costly and as a consequence, few studies have been able to examine spatiotemporal changes in distribution and abundance of marine debris over a large area (but see Cózar et al.^1^, Eriksen et al.^27^). Moreover, there is even less information available on the spatial overlap between the distribution of marine debris and that of marine wildlife at risk from this debris. Yet, quantifying this overlap is essential to identify those areas where threats related to ingestion and entanglement at sea are the most acute for marine megafauna^25^.

Among seabirds, albatrosses (family Diomedeidae) comprise 22 species, of which 17 are globally threatened according to the IUCN Red List^28^. Albatrosses are frequent scavengers, and accordingly they regularly happen to ingest plastic debris, thereby increasing their mortality rates^29,30^. Studying the foraging ecology of albatrosses has benefited considerably from the development of tracking techniques via satellites over the past three decades, showing that albatrosses can travel hundreds of kilometers in a few days to locate their food in the open ocean^31,32^. More recently, animal-borne image loggers have allowed researchers to further examine the context of these ingestions of food and debris, and account for proximate factors such as interactions with other predators, or the nearby presence of fishing vessels^33–35^. Therefore, the simultaneous deployment of both GPS- and video-loggers on albatrosses is expected to conveniently provide new insight into the spatial distribution of floating large debris across vast oceanic areas, and on the behavioral response of the birds encountering these debris. Marine top predators such as seabirds do not distribute uniformly at sea, and their distributions are affected by physical and biological factors operating at various scales^36,37^. Thus, although albatrosses are not an unbiased sampler for the debris distribution, this seabird-based approach allows to compare the relative distribution of debris between their foraging areas.

In the North Pacific Ocean, black-footed albatrosses (*Phoebastria nigripes*) breeding on Torishima (Japan) forage near the Izu chain over the Kuroshio Current area^38^. The Kuroshio Current, flowing northward, has also been shown to play an important role in transporting plastics across the North Pacific Ocean^22,24^. Therefore, the spatial distribution of floating debris in this region is expected to overlap with the foraging range of the black-footed albatrosses from Torishima.

In this paper, we aimed at determining the distribution of floating debris in the north-western Pacific Ocean, using a combination of video and GPS data loggers deployed on free-ranging black-footed albatrosses breeding on Torishima. In addition, we also quantified the spatial overlap between the debris distribution and the albatrosses’ foraging areas, and examined the behavioral response of the birds towards the encountered debris.

## Results

**Figure 1:**
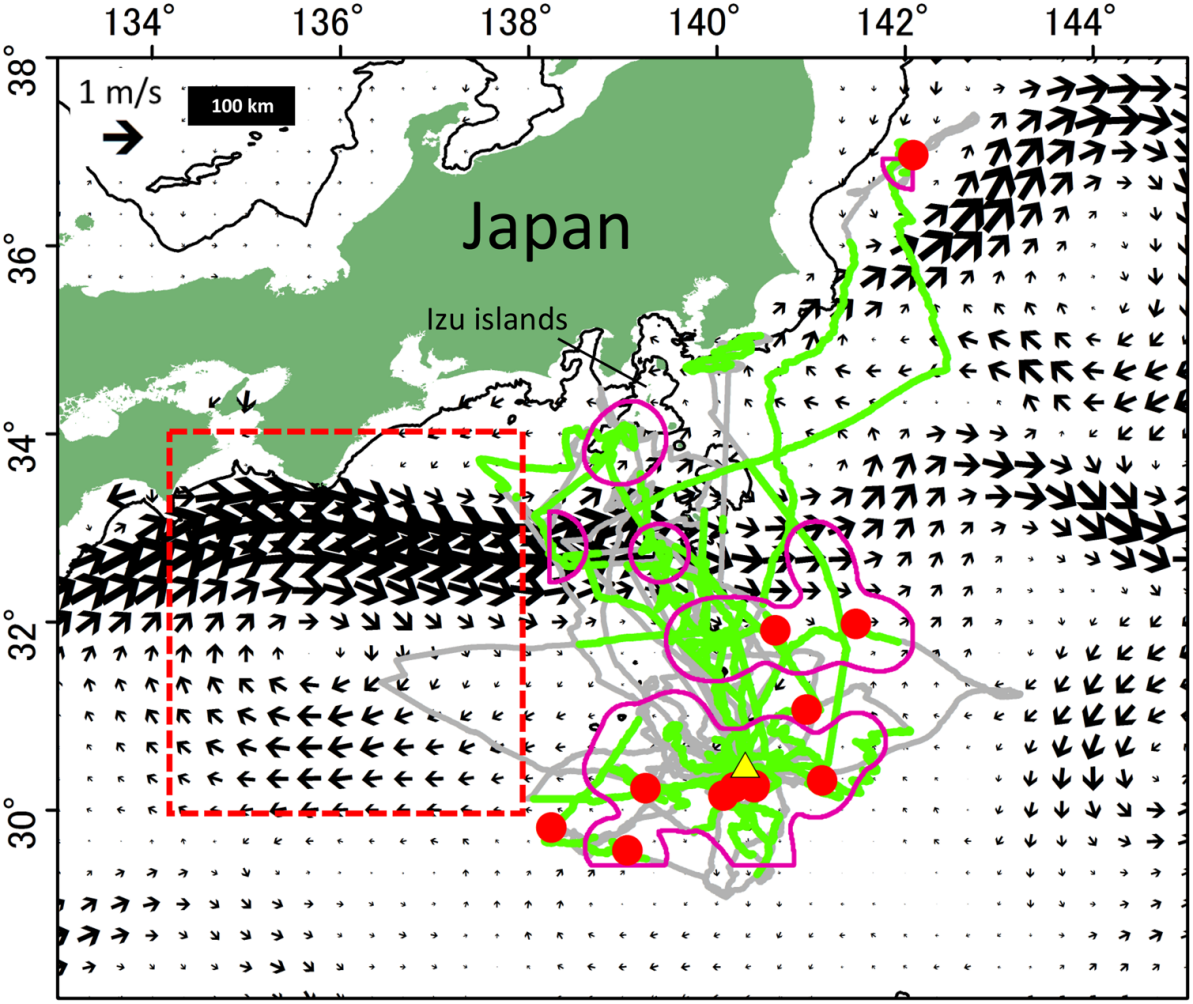
Distribution of marine floating debris (red circles) encountered by black-footed albatrosses at sea in relation to their foraging area (purple lines: 95% kernel density contour of the on-water bout locations where the birds fed on squid or fish confirmed from video footage). GPS tracks of 23 foraging trips made by 13 birds from Torishima (yellow triangle) are shown by gray lines, with green sections symbolizing when concomitant video records were available. Black arrows show surface ocean current during 15 February–2 March 2017, when field survey was conducted. Isobaths of 500 m are shown by black line. Red square represents study area of Yamashita and Tanimura (2007) where small floating plastic samples were collected using a neuston net over the Kuroshio Current. The figure was generated with the ArcGIS Desktop 10.7.1 software (https://www.esri.com/en-us/home).

We obtained both GPS and video data adequately from 13 birds that made 23 trips at sea (Table S1). These trips extended to 247.7 ± 185.9 km and lasted 34.7 ± 21.3 h, on average (Table S2). Across these foraging trips, black-footed albatrosses distributed mainly in the areas north of their colony along the Izu Chain (Fig. 1), covering distances of 985.2 ± 705.2 km during each trip, on average.

**Figure 2:**
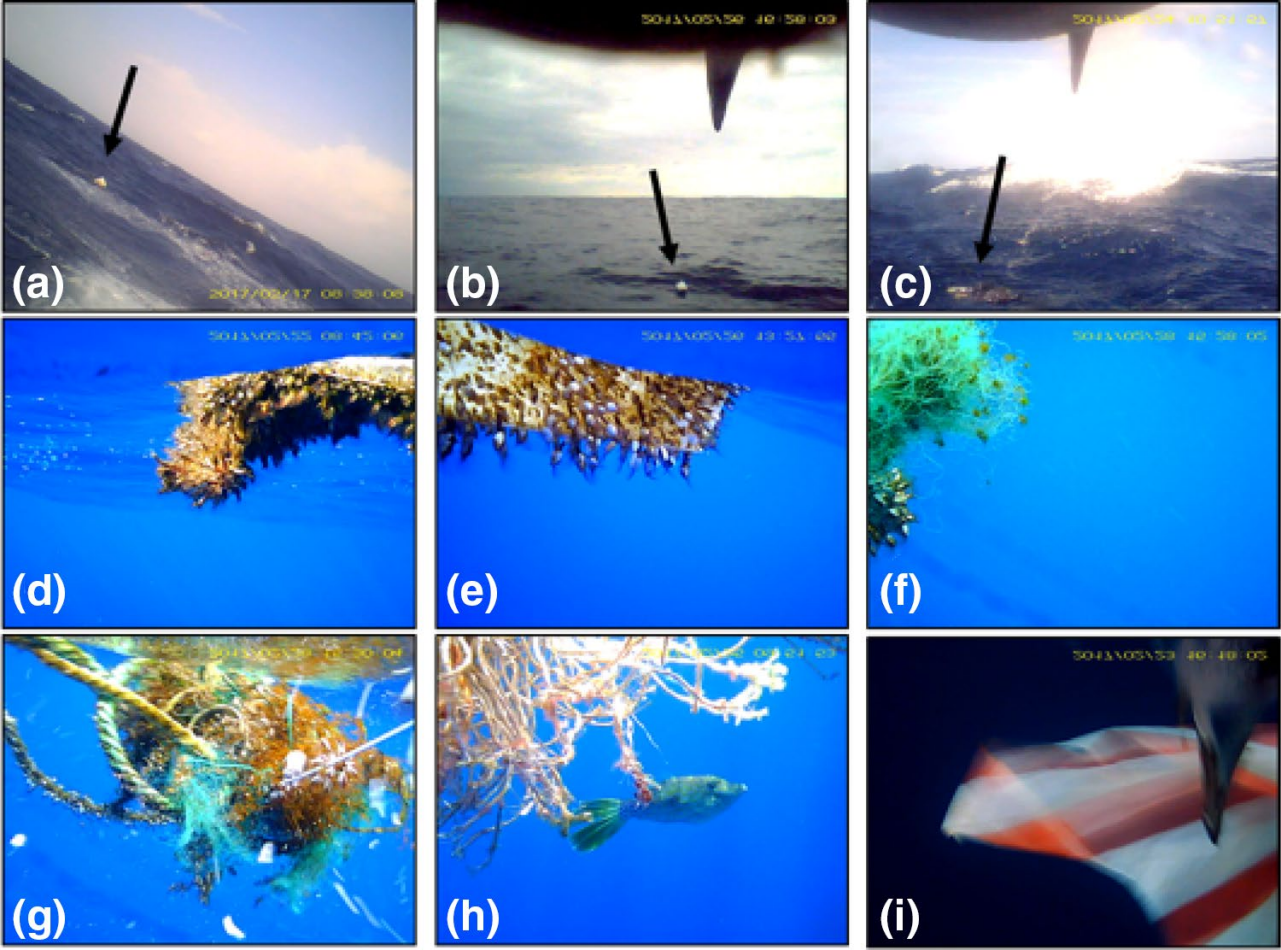
Examples of marine debris floating at the sea surface, recorded by video-loggers fitted on black-footed albatrosses breeding on Torishima, Japan. Styrofoam recorded from Bird 31 (**a**), styrofoam, Bird 48 (**b**), plastic, Bird 39 (**c**), plastic with barnacles, Bird 42 (**d**), styrofoam with barnacles, Bird 46 (**e**), fishing net with barnacles, Bird 49 (**f**), fishing net and rope with barnacles, Bird 49 (**g**), rope used in fisheries, harboring a *Aluterus scriptus* filefish, Bird 43 (**h**), and Bird 44 pecking at a plastic sheet (**i**). Images were extracted from footage taken during flight bouts (**a**–**c**), or during sitting-on-water bouts (**d**–**i**).

From 22 trips of 13 birds (Table S2), we were able to collect 8492 video clips (7674 in flight, 818 on water), taken every 2 min during the daytime and each one lasting 3 s (see methods). A total of 16 floating debris items (visible on 26 videos) were recorded from 9 trips of 9 birds (Table S2). Of these, five debris items (from 4 videos) were recorded during flight, and 11 others (from 22 videos) while the birds were sitting on the water (Table S2). The debris encountered by the albatrosses included styrofoam (n = 4), plastic pieces (n = 3), plastic sheet (n = 1), fishery ropes (n = 2), fishery netting (n = 1), fishery netting with ropes (n = 1), unidentified debris items (n = 4) (Fig. 2). Detailed examination of the footage revealed that most of the debris had barnacles and seaweeds attached to them (Fig. 2d–g). We also observed a bird pecking at a plastic sheet with red-white stripes while sitting on the water, but the video was too short to know whether the bird ate it or not (Fig. 2i).

**Figure 3:**
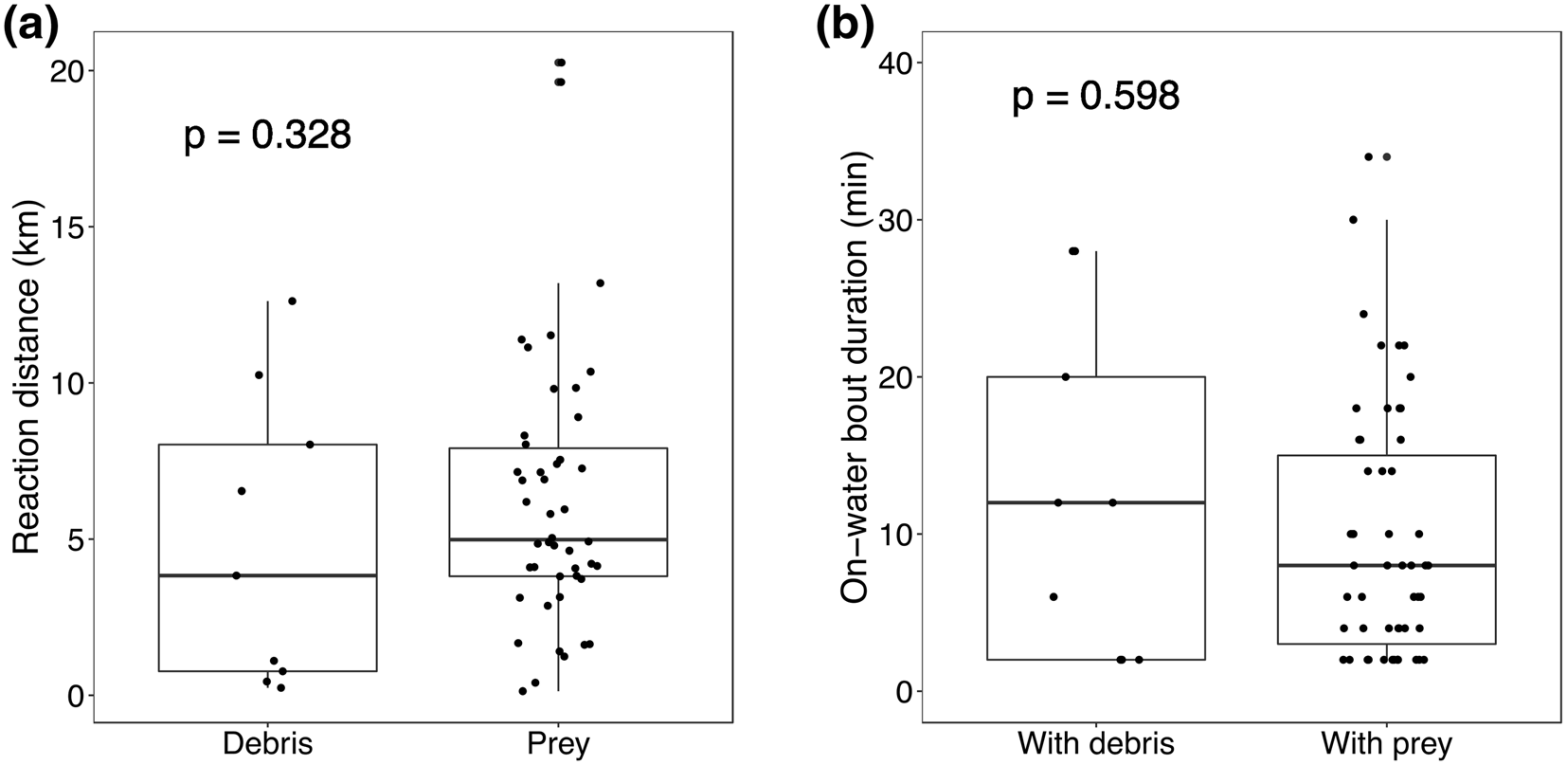
Comparison of the behavioral response of black-footed albatrosses to debris and prey. Reaction distance toward debris (n = 9) and prey (n = 46) (**a**). On-water bout durations between those with debris (n = 9) and prey (n = 61) (**b**). Results of Mann–Whitney *U* test are shown in each panel.

Break-point analysis on trajectories (see methods) showed that black-footed albatrosses changed their flight course toward debris at a mean distance of 4.9 ± 4.7 km (0.2–12.6 km, n = 9) from the debris. This reaction distance was similar to the case when albatrosses approached toward prey (6.3 ± 4.3 km, 0.1–20.3 km, n = 46) (Mann–Whitney *U* test, p = 0.328) (Fig. 3a). Further, the duration of on-water bouts was not different between those with debris (12.4 ± 10.7 min, 2.0–28.0 min, n = 9) versus prey (9.6 ± 7.9 min, 2–34 min, n = 61) (Mann–Whitney *U* test, p = 0.598) (Fig. 3b). These results indicate that black-footed albatrosses behaviorally respond to debris as they would respond to their natural prey, including squids and fish.

We identified six foraging areas of black-footed albatrosses from kernel density estimation contours using locations where the birds fed on natural prey confirmed by video data (Figs. 1 and S1, see methods). Two of six foraging areas had debris, and the rate of debris encounter (debris abundance divided by the area covered estimated from a distance traveled and reaction distance, see methods) within the foraging area near Torishima was higher (0.05 debris item/ 100 km^2^) than in the other area (0.03 debris item/ 100 km^2^) (Fig. 1). These areas were characterized by weak surface current (Figs. 1 and S2) and distributed south of the Kuroshio Current, which was flowing eastward at high velocities (> 1 m/s).

## Discussion

To our knowledge this is the first study to provide (1) the distribution of large marine debris within the foraging range of free-ranging oceanic seabirds and (2) their behavioral responses to debris at sea. This achievement is particularly relevant as the bio-indicating species studied here (black-footed albatross) is one of the marine organisms known to interact the most frequently with anthropogenic debris at sea.

Foraging areas of black-footed albatrosses were widely distributed over oceanic habitats, and higher debris encounter rates occurred in the foraging areas located near Torishima, where the surface current was relatively weak. These foraging areas with relatively high densities of debris might therefore represent a high risk of exposure to debris. In our study area in the North Pacific Ocean, the Kuroshio Current is a major source in transporting debris including plastics^22,24^. Although it was unclear from our study which were the physical processes involved in the high encounter rate of debris observed near Torishima, the slower currents might contribute to the increased local persistence of floating debris at the sea surface, resulting in debris accumulation and/or better detection by flying seabirds including black-footed albatrosses.

In our study, although it was not possible to determine the exact size of the encountered debris, we estimated that they were in the range of tens of centimeters, using the bill size of black-footed albatrosses (ca. 100 mm) and the fish visible on the footage (*Aluterus scriptus*, common length 55 cm^39^) as references, when feasible. These items were thus comparable in size to debris found in previous, ship-based direct observations conducted in other regions^25,40,41^. Floating plastic pieces previously collected by a neuston net with a 330 μm mesh size in the Kuroshio Current area (upstream of our study region, Fig. 1) ranged 1–280 mm in size, with the 3 mm size-class being predominant^24^. At Kure Atoll, 4000 km east of Torishima, black-footed albatrosses were found to ingest such small plastic debris, predominantly in the range 5–25 mm^42^. However, such small debris items were not detected in our study, presumably because the resolution of video images was too low, especially in the distance.

Break-point analyses suggested that black-footed albatrosses might approach the debris similarly to when they scavenge for food^35^. We also found that reaction distances to both debris and prey varied greatly (between 0.1 and 20 km), suggesting that albatrosses use both visual and olfactory cues to locate floating debris. Most of the floating debris encountered by black-footed albatrosses were encrusted with fauna including barnacles and seaweeds that can produce infochemicals (e.g. dimethyl sulfide, DMS), to which foraging seabirds including albatrosses are attracted from tens of kilometer away^43,44^. Once the birds come closer to debris, the actual decision to sit nearby and eat debris is more likely to become based on vision. Indeed, the studied albatrosses did not peck most of the debris items they encountered but pecked a plastic sheet with red-white stripes. This observation fits in with that the seabirds that perceive debris items from above are more prone to ingest light color items^45^, although our sample size was too small to conclude what type of debris more attracts and is ingested by seabirds. Another explanation on why floating debris attracts seabirds is that the birds feed on aggregated prey associated with debris. Fish may often aggregate in great numbers under floating objects for some reasons, including protection from predators, feeding on invertebrates attached to the debris, spawning substrates, and others^46^. In black-footed albatrosses, the birds are known to feed substantially on eggs of flying-fish^47^, and flying-fish spawn on floating substrata including both vegetal and artificial debris^48^.

Seabird-debris interactions can severely reduce the birds’ survival through ingestion and/or entanglement^11,12,14^. In fact, black-footed albatrosses showed high prevalence rates of plastic ingestion (45–100% occurrence^30^) among adult individuals by-caught in the eastern North Pacific. Necropsies of dead specimens from the Northwestern Hawaiian Islands also revealed high plastic ingestion rates (96.4% in chicks and 58.8% in adults^49^), indicating that marine debris constitutes an additional threat at sea for black-footed albatrosses’ survival. Moreover, plastics ingested by black-footed albatrosses breeding on Mukojima, Japan, contain hazardous chemical additives^19^. At Torishima, more than 50% of the monitored nests of this species contained fishing gear (lines, snoods, hooks)^38^. Our study further highlights that black-footed albatrosses breeding on Torishima recurrently interact at sea with floating debris related to human activities, and the fact that these debris items occurred predominantly within the foraging areas of the albatrosses increases the risk of ingestion or entanglement by the foraging birds. In addition, we found that once the albatrosses were attracted to floating debris, they spent as much time manipulating debris as feeding on natural prey such as squid and fish, which may lead to reduced energy gain in a given foraging trip.

In conclusion, we were able to determine the distribution of large debris floating at the ocean surface within the foraging ranges of wide-ranging oceanic seabirds, using a combination of GPS- and video-loggers deployed directly on the birds. We also provided behavioral responses of seabirds to large debris floating on the ocean. Our approach based on albatrosses to monitor debris at sea can therefore be used to quantify the degree of exposure to seabirds among their foraging areas and to examine the nature of the interactions between seabirds and large floating debris: for example, what type of debris may predominantly attract the predators and be ingested. As plastic pollution is likely to continue increasing in the oceans and further threatening marine wildlife for the decades to come, it will become critical to monitor vast areas of the open oceans to understand the spreading and severity of this issue: a task that will only become more logistically demanding using conventional approaches. Taking advantage of the increasing use of relatively inexpensive bio-logging techniques worldwide, we show that large-sized and wide-ranging predators can be used to conveniently monitor, understand and predict the distribution of large marine debris across the world’s oceans, and their impact on marine wildlife.

## Methods

### Field study

Field work was carried out at the black-footed albatross colony on Torishima (30.48°N, 140.30°E), Japan during 16 February-5 March 2017. We instrumented 17 birds rearing chicks with a GPS-logger (GiPSy5, TechnoSmart, Montecelio, Italy, 45 × 22 × 18 mm, 17 g with a 600 mAh battery) on the back and a video-logger (DVL400M065-IB, Little Leonardo, Tokyo, Japan, 61 × 21 × 15 mm, 29 g, 1280 × 960 resolution, 30 frames/s, 43° vertical angle of view, 33° horizontal angle of view) on either the back (for 4 birds brooding chicks) or belly (for 13 birds rearing larger chicks) with Tesa tape (Table S1, Fig. S3). GPS positions were recorded every 20 s, and 3-s long videos were recorded every 2 min during daytime (7:00–17:00 local time). A time stamp was programed to appear on each 3-s long footage, showing local date and time.

To avoid disturbing the colony with frequent visits to the study nests for recapturing the tagged birds, we also attached a VHF transmitter (Sakura transmitter LT-04-02, Circuit Design, Inc., Nagano, Japan, 150 MHz, 30 × 11 × 9 mm with 150 mm, 5 g) on each tagged bird. The signal (150 MHz) of transmitters was received from > 500 m on a fixed antenna.

All field experimental protocols were approved by the Japan Ministry of the Environment and Agency for Cultural Affairs, and Tokyo Metropolitan Government. All methods were carried out in accordance with relevant guidelines and regulations, including animal ethics approvals from Hokkaido University, Japan. Also, the study was carried out in compliance with the ARRIVE guidelines.

### Analysis

GPS data were analyzed following Thiebot et al.^38^. After removing all positions at the colony, we linearly interpolated positions at 1 min intervals. We used footage obtained from video data loggers to determine the presence of debris, the debris type, and the bird activity (i.e., in flight or on the water), and carefully checked each 3-s footage (frame by frame) by eye. GPS and video data were matched based on time records, and we defined an ‘on-water bout’ as all consecutive GPS positions with the corresponding 3-s video clips showing uninterrupted sitting-on-water behavior, and a ‘flight bout’ as consecutive positions corresponding to flight activity. In the case of birds with video-loggers attached on the belly, footage during both flight and sitting on the water was analyzed, while in the case of birds with video-loggers on the back, only the footage recorded during flight was used, because the water surface was not visible from the back of birds sitting on the water. Unclear footage during flight was not used for the analysis (Table S2). There was no unclear footage recorded while birds were sitting on the water (Table S2).

To compare reaction distances toward debris or prey (i.e., when the birds modified their behavior upon encountering them), we tested for a significant breaking point along the flight bout < 30 min before encountering either debris or prey (i.e., squid or fish), respectively, using a line simplification algorithm, the Douglas–Peucker algorithm^50,51^ using “kmlShape” package in R software (version 4.0.2^52^). This algorithm was designed to reduce the number of points in a line by removing the ones that produce little variation around a more direct simplified line. For this analysis we excluded debris and prey recorded by birds during flight because those recorded were passed by flying birds and used on-water bouts with debris items (n = 9) and with prey (n = 46) collected from birds with video-loggers on the belly. Then we compared the reaction distances between debris and prey using Mann–Whitney *U* tests. Further, we compared the duration of on-water bouts between those with debris (n = 9) and with prey (squids or fish) (n = 61) using Mann–Whitney *U* test.

To examine the effect of surface currents, including the Kuroshio Current, on the debris distribution we used the online portal for surface current data (Ocean Surface Current Analysis Real Time, https://podaac.jpl.nasa.gov/dataset/OSCAR_L4_OC_third-deg) during the same period of our field survey. The spatial and temporal resolutions of the current data were 0.33° degree and five days, respectively.

To investigate spatial overlap between the debris distribution and the albatross foraging areas, we defined the foraging areas as the 95% kernel density estimation contours computed from only locations where the albatrosses fed on natural prey (squids or fish) confirmed from video footage (i.e., 61 on-water bouts with prey, Table S2, Fig. S1) with 0.5° of search radius using Spatial Analyst tool in ArcGIS 10.7.1. We then compared debris encounter rate among foraging areas of the albatrosses as follows; we calculated debris encounter rate within each foraging area, as plastic abundance divided by area covered. The area covered was estimated using distances travelled and mean reaction distance identified by break point analyses. The relationship between debris encounter rate within a foraging area of black-footed albatrosses and surface current speed was examined using the exponential regression analysis.

## Supplementary Information

Supplementary Information.
