# Supplementary material for: Mapping marine debris encountered by albatrosses tracked over oceanic waters
Source: Sci Rep. 2021 May 25;11:10944. doi: 10.1038/s41598-021-90417-x (PMC8149674; doi:10.1038/s41598-021-90417-x)
Supplement: Supplementary file 1 — Supplementary Information. [file 41598_2021_90417_MOESM1_ESM.pdf]

Supplementary information from “**Mapping marine debris encountered by albatrosses tracked over oceanic waters**”

Bungo Nishizawa*, Jean-Baptiste Thiebot, Fumio Sato, Naoki Tomita, Ken Yoda, Rei Yamashita, Hideshige Takada, Yutaka Watanuki

*Corresponding author: nishizawa@salmon.fish.hokudai.ac.jp

Table S1. List of sampled birds. Bird ID, sex, video on back or belly, video recording interval, GPS sampling interval, video data (Y: Yes, N: No), GPS data (Y: Yes, N: No), both video and GPS data (Y: Yes, N: No), date and time of deployment and retrieval are shown.

Bird ID	Sex	Video on	Video interval	GPS interval	Video data	GPS data	Both video and GPS data	Deployment (local time)	Retrieval (local time)	Note
31	F	back	2 min	20 sec	Y	Y	Y	2017/2/17 12:43	2017/2/19 13:10	
32	M	back	2 min	20 sec	Y	Y	Y	2017/2/17 14:12	2017/2/22 12:49	
33	F	back	2 min	20 sec	Y	Y	Y	2017/2/17 14:47	2017/2/22 16:51	
34	M	back	2 min	20 sec	Y	Y	Y	2017/2/17 15:42	2017/2/19 14:35	
35	F	belly	2 min	20 sec	N	Y	N	2017/2/17 16:17	2017/2/20 12:12	video error
39	F	belly	2 min	20 sec	Y	Y	Y	2017/2/20 9:10	2017/2/25 13:55	
40	U	belly	2 min	20 sec	N	N	N	2017/2/20 10:29	NA	not recaptured
41	M	belly	2 min	20 sec	Y	Y	Y	2017/2/21 11:27	2017/2/23 9:54	
42	F	belly	2 min	20 sec	Y	Y	Y	2017/2/21 12:00	2017/2/26 11:04	
43	M	belly	2 min	20 sec	Y	Y	Y	2017/2/22 9:25	2017/2/26 10:05	
44	F	belly	2 min	20 sec	Y	Y	Y	2017/2/22 10:42	2017/2/25 10:04	
45	U	belly	2 min	20 sec	N	N	N	2017/2/23 8:37	NA	not recaptured
46	M	belly	2 min	20 sec	Y	Y	Y	2017/2/23 9:15	2017/2/26 16:08	
47	U	belly	2 min	20 sec	N	N	N	2017/2/23 15:38	NA	not recaptured
48	F	belly	2 min	20 sec	Y	Y	Y	2017/2/25 15:04	2017/2/28 10:10	
49	F	belly	2 min	20 sec	Y	Y	Y	2017/2/26 8:36	2017/3/1 15:20	
50	M	belly	2 min	20 sec	Y	Y	Y	2017/2/26 15:00	2017/3/1 8:00	

Table S2. Trip metrics and number of video footage, number of video footage with debris, number of debris either during flying or during sitting on water, and number of on-water bout with prey.

Bird ID	Trip ID	Maximum range (km)	Total distance (km)	Duration (h)	Video on	N of 3-s video footage during flying (including unclear)	N of unclear 3-s video footage during flying	N of 3-s video footage during sitting on water	N of 3-s video footage during flying with debris	N of 3-s video footage during sitting on water with debris	N of debris during flying	N of debris during sitting on water	N of on-water bout with prey
31	1	239.5	1067.6	43.7	back	363	2	-	1	-	1	0	1
32	2	262.5	684.9	27.7	back	260	0	-	0	-	0	0	0
32	3	49.6	181.2	17.8	back	152	2	-	0	-	0	0	1
32	4	283.5	777.0	42.3	back	340	0	-	0	-	0	0	0
32	5	225.4	583.6	22.9	back	208	0	-	0	-	0	0	0
33	6*	6.5	33.4	2.1	back	-	-	-	-	-	-	-	-
33	7	416.8	1351.9	38.6	back	305	31	-	0	-	0	0	2
33	8	5.5	19.6	0.6	back	12	0	-	0	-	0	0	0
33	9	505.2	1962.5	57.7	back	555	7	-	0	-	0	0	2
34	10	303.8	879.6	44.2	back	400	1	-	0	-	0	0	0
39	15	424.5	1560.5	43.3	belly	588	8	242	1	0	2	0	8
41	16	98.7	611.4	21.2	belly	178	0	32	0	0	0	0	7
42	17	293.4	859.3	24.7	belly	220	0	34	0	1	0	1	6
42	18	465.2	2294.3	73.5	belly	840	2	84	0	0	0	0	6
42	19	39.4	138.7	4.8	belly	99	0	15	0	0	0	0	3
43	20	26.6	198.9	5.9	belly	162	0	16	0	0	0	0	1
43	21	223.4	1024.7	41.3	belly	335	81	67	0	2	0	1	1
44	22	797.2	2740.8	63.3	belly	636	0	2	0	1	0	1	1
46	23	362.2	1206.5	27.6	belly	192	0	14	0	0	0	0	2
46	24	152.4	539.3	17.6	belly	207	0	50	0	11	0	2	4
48	25	174.4	1277.7	50.1	belly	583	0	34	1	1	1	1	5
49	26	201.9	1755.6	76.3	belly	718	1	71	0	4	0	3	7
50	27	140.0	910.3	50.3	belly	460	4	157	1	2	1	2	4
**Mean**		**247.7**	**985.2**	**34.7**	**Sum**	**7813**	**139**	**818**	**4**	**22**	**5**	**11**	**61**

* Video data were not obtained in this trip

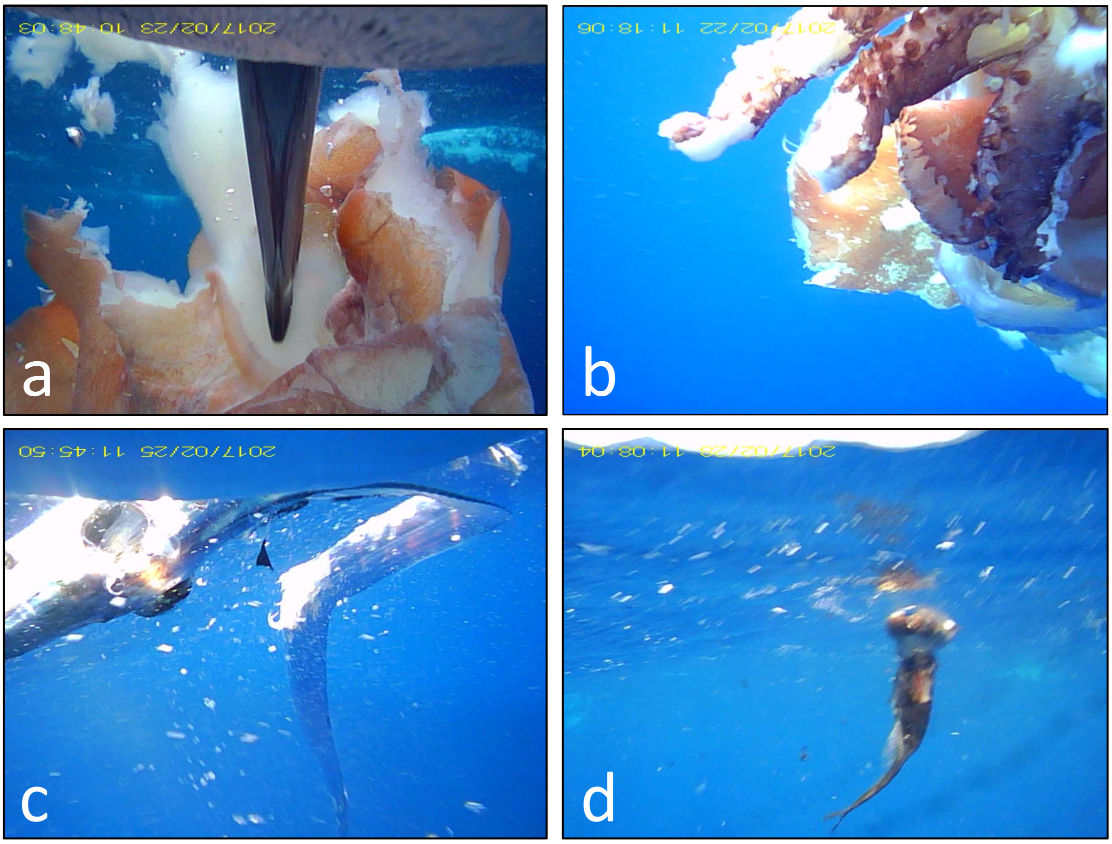

Figure S1. Examples of prey recorded by video-loggers fitted on black-footed albatrosses breeding on Torishima, Japan. Squid recorded from Bird 41 (a) and Bird 43 (b). Fish recorded from Bird 42 (c) and Bird 49 (d). Images were extracted from footage taken during sitting-on-water bouts.

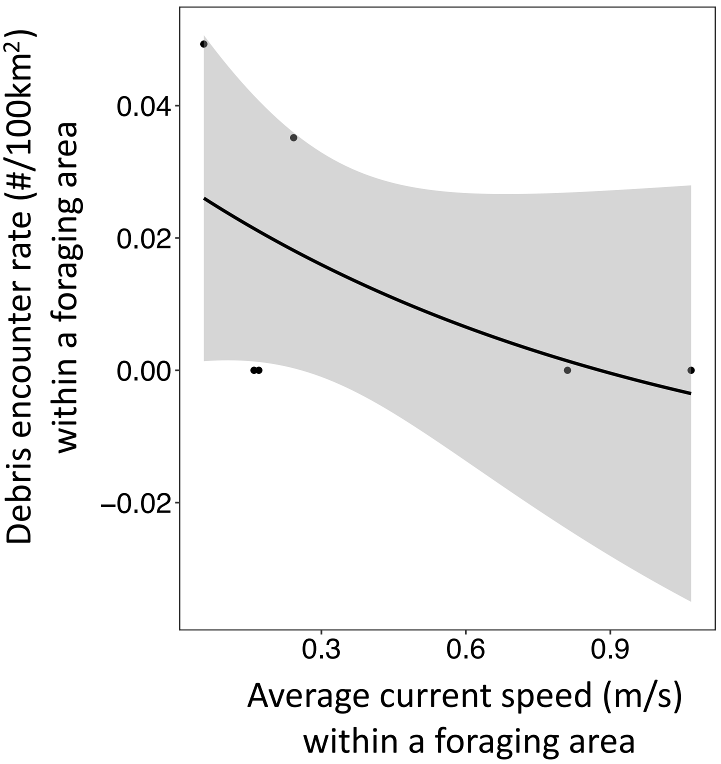

Figure S2. The relationship between debris encounter rate within a foraging area of black-footed albatrosses and average current speed within a foraging area. Each point shows a different foraging area defined as kernel density estimation counters using locations where the birds fed on natural prey, including squids and fish, confirmed by video footages (see methods). The solid line shows the exponential regression line with 95% confidence intervals.

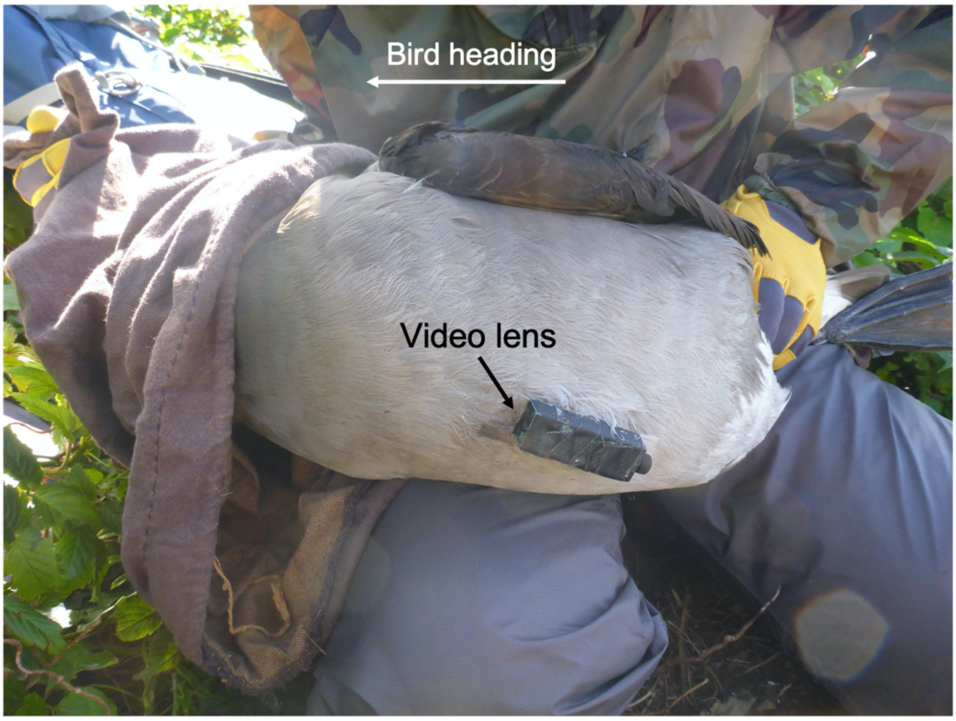

Figure S3. A black-footed albatross attached a video-loggers (61×21×15 mm, 29 g) on the belly with Tesa tape.
